# Proximity to Major Roads and Risks of Childhood Recurrent Wheeze and Asthma in a Severe Bronchiolitis Cohort

**DOI:** 10.3390/ijerph18084197

**Published:** 2021-04-15

**Authors:** Rachel D. Freid, Ying (Shelly) Qi, Janice A. Espinola, Rebecca E. Cash, Zahra Aryan, Ashley F. Sullivan, Carlos A. Camargo

**Affiliations:** Department of Emergency Medicine, Massachusetts General Hospital, Harvard Medical School, Boston, MA 02114, USA; rachel.freid75@gmail.com (R.D.F.); YQI6@mgh.harvard.edu (Y.Q.); ESPINOLA@helix.mgh.harvard.edu (J.A.E.); RCASH@mgh.harvard.edu (R.E.C.); ZARYAN@bwh.harvard.edu (Z.A.); afsullivan@partners.org (A.F.S.)

**Keywords:** prospective cohort, children, bronchiolitis, geographic information systems, major roads, traffic, air pollution, recurrent wheeze, asthma

## Abstract

Air pollution exposures have been suggested as risk factors for childhood respiratory diseases. We investigated proximity to major roads, an indicator of air pollution exposure, and its associations with childhood recurrent wheeze and asthma. We used data from a multicenter prospective cohort study of 921 infants hospitalized for bronchiolitis and recruited from 14 U.S. states. Primary exposure was residential proximity to the nearest major road at birth through age 3 years. Residential distance from nearest major road was divided into four categories: <100, 100–200, 201–300, and >300 m. Outcomes were parent-reported recurrent wheeze by age 3 years and asthma by age 5 years. Associations between residential proximity to major roads and respiratory outcomes were investigated using multivariable Cox proportional hazards modeling and logistic regression, adjusted for confounders. Out of 920 participants with home address data, pooled estimates identified 241 (26%) participants resided within 300 m of a major road, 296 (32%) developed recurrent wheeze by age 3, and 235 out of 858 participants (27%) developed asthma by 5 years. Participants who resided close to a major road had the highest risk of recurrent wheeze (adjusted hazards ratio for <100 m, 1.59, 95%CI: 1.08–2.33) and asthma (adjusted odds ratio for 201–300 m, 1.62, 95%CI: 1.16–2.25), compared to those residing >300 m from a major road. Proximity to major roads is associated with increased risks of recurrent wheeze and asthma in young children.

## 1. Introduction

Respiratory problems in young children are highly prevalent and, in particular, recurrent wheeze and asthma are a significant worldwide public health issue [1,2,3,4,5]. Wheezing episodes are common in young children and an estimated 50% experience at least 1 wheezing episode between the first year of life and age 6 years [4,6,7]. Previous literature reports that severe and frequent wheezing episodes during the first 3 years of life may herald early manifestations of asthma [3,8]. Indeed, asthma is the most common non-communicable disease and accounts for over 5 million respiratory illnesses in children [3,4,5,9]. Although asthma diagnoses are typically made in school-aged children, most symptoms of asthma begin during infancy [3,10]. A growing body of evidence suggests that both recurrent wheeze and asthma are multifactorial disorders, necessitating investigation into possible early-life influences with an important focus on environmental factors.

The health effects of air pollution on the general population remains a worldwide public health concern and infants and young children are particularly vulnerable [2,11]. Infants and young children have elevated air inhalation, respiratory rates, and baseline metabolic rates compared to older individuals [11]. In addition, infants and young children are more susceptible to the effects of air pollutants as the first 3 years of life are critical for immune system and airway development [11,12]. It is widely recognized that air quality plays a crucial role in early-life development and exacerbation of airway disease, including asthma [2,5,13]. Of interest is traffic-related air pollution (TRAP) where researchers have previously shown that TRAP exposure during infancy is associated with decreased lung function and long-term respiratory consequences in allergen- and inhalant- sensitive children [2,14]. Decreased lung function has also been associated with increased risk for developing asthma [15]. Earlier studies note that infants and young children exposed to TRAP is a suggested risk factor for childhood asthma [16,17]. However, positive associations between air pollution and recurrent wheeze or asthma remain inconclusive and may be in part to heterogeneity of study methods [16,18,19,20,21,22,23]. Further, limited studies have assessed whether TRAP exposure increases risk of adverse respiratory health effects in children that have experienced respiratory stress, such as severe bronchiolitis, earlier in life [24]. One such study has reported an association between TRAP and increased asthma, though insignificant for wheezing, among children with bronchiolitis; however, this study assessed children residing in seven major cities across Korea [24]. Combined, these studies add to a body of evidence suggesting the importance of studying sensitive or high-risk pediatric populations when evaluating the risk of chronic respiratory disease, including asthma [2,14,24,25].

Residential proximity to major roads (high traffic roads) is commonly used as a surrogate measure of exposure to TRAP [17,18]. While previous prospective cohort studies have focused on specific air pollutants (such as nitrogen dioxide), real environment exposures to other pollutants and traffic-related stressors are inevitable [2,17,18,26]. It is plausible that combined exposure to multiple types of TRAP act synergistically, thereby causing a more hazardous effect compared to analyzing only 1 traffic-related air pollutant [26]. To assess the association of broad TRAP exposure and asthma incidence in school-aged children, earlier studies have evaluated this association yet only at specific time points [16,27]. Further longitudinal, cumulative studies are necessary to evaluate the association of early-life TRAP exposure, as measured by residential proximity to TRAP sources, and development of recurrent wheeze and subsequent asthma in susceptible populations [14,17,24,25]. Our study aims to investigate the association of cumulative early-life residential proximity to the nearest major or primary road with risk of recurrent wheeze at birth to age 3 years and incidence of asthma by age 5 years among children with a history of severe bronchiolitis at infancy.

## 2. Materials and Methods

### 2.1. Study Population and Data Collection

This study is a secondary analysis of data from the Multicenter Airway Research Collaboration-35 study (MARC-35) cohort. The primary goal of MARC-35 study was to investigate infectious etiology of a child’s severe bronchiolitis, and subsequent development of recurrent wheeze by age 3 years and asthma by age 5 years. More details on the original cohort design, participants recruitment, general characteristics and primary results can be found elsewhere [28]. Briefly, participants were recruited from 17 sites during fall/winter of 2011–2014 (Table S1). The coordinating center is located at the Emergency Medicine Network (EMNet; www.emnet-usa.org), based at Massachusetts General Hospital (Boston, MA). Infants (age < 1 year) hospitalized for bronchiolitis were enrolled in MARC-35. Exclusion criteria were certain chronic conditions (e.g., known heart-lung disease, immunodeficiency) or gestational age < 32 weeks. Consent was obtained from all parents/guardians and was translated into Spanish as needed. Institutional review boards approved the study protocol at all recruitment sites.

Participant baseline data were predominantly collected through standardized parent and/or legal guardian interviews conducted at enrollment. Parent-reported variables included participant’s demographics, medical, family, and environmental history as well as details of acute illness [28]. Medical record data were also abstracted by enrolling sites, providing emergency department and inpatient clinical information regarding the index bronchiolitis admission [28]. We asked parents for the residential (home) addresses of participants and dates of moves starting from birth. Their addresses were updated and confirmed during follow-up phone calls which were conducted biannually. All addresses were subsequently validated by site coordinators through Google maps and confirmed through successful delivery of postal packages sent from the coordinating center to each participant’s address. In case of discrepancy between postal address and residential address of child, the latter was used for exposure measurements. In addition, we excluded participants who refused to disclose exact residential location or only provided post office (PO) boxes as contact addresses. In this analysis, we include all residential addresses up until age 3 years of each participant. During data processing, all addresses were geocoded using ArcGIS Pro 2.7 (ESRI, Redlands, CA, USA). Separate vector layers were created for each movement address and birth address. Participants’ family income information was estimated using ZIP code at enrollment using median household income estimated from ZIP code. Population density of each neighborhood the participant lived was estimated using 2010 census block geographic data with center of population at each census block from the US Census Bureau.

The location of major and primary roads was obtained from the 2015 Topologically Integrated Geographic Encoding and Referencing (TIGER)/Line shapefiles published by the US Census Bureau. The 2015 road shapefile was chosen since it was the year that most participants stopped moving. Major roads, including both primary and secondary roads, were defined as roads with annual average total traffic count ≥10,000 vehicles per day. Primary roads were defined as interstate or state highways with average total traffic count ≥30,000 vehicles per day. Primary roads are distinguished by the presence of interchanges and speed limit of >50 miles per hour. These geocoded road shapefiles were used to obtain distance to the nearest primary road and major road from residential address of each participant in meters. Major road and primary density from each participant residential address within a 250-m radius of a circular buffer area were calculated using the road shapefiles to be in line with previous studies [18]. In addition, length of major roads (km) in this buffer area was summed and used to calculate major road density (total km roadways per km^2^ land). 

### 2.2. Exposure Assessment

The primary exposure measure of this study was the proximity to the nearest major road from participants’ residential addresses. Previous studies showed that TRAP concentration decreases with increasing distance from TRAP sources, and variables such as length of high traffic roads or traffic density within 100–300 m can explain spatial variation in TRAP [19,29,30,31]. Thus, we considered maximum exposure in those who lived <100 m from a major road, followed by living within 100–200 and 201–300 m from a major road. Participants who lived >300 m to the nearest major road were considered as unexposed and thus used as the reference group in multivariable analysis. The secondary exposure of interest in this study was proximity from participants’ residential addresses to the nearest primary road. We categorized each participant’s residential proximity to primary roads into 3 groups: <400, 401–1000, and >1000 m. Those who resided >1000 m of a primary road were considered as unexposed and used as the reference group in unadjusted and multivariable analysis.

The number of residential moves for each participant varied from 1 to 6, including birth address. A weighted model according to length of stay in each address was used to pool exposures. The following formula was used to calculate the pooled exposure:

(1) ∑i=16=Di·Ei∑i=16Di

where “*D*” denotes duration spent in address “*i*” before age 3 years, and “*E*” is the pooled exposure measurement from that address, in this case, the proximity to major road, primary road, and also population density in the residential area. For cases of recurrent wheeze by age 3 years, we accounted for each participant’s address and duration of stay until the day they developed the recurrent wheeze outcome; thus, “*D_i_*” is either the day when the recurrent wheeze outcome had been achieved or, for non-cases, until the last date of the child’s observation period up to age 3 years. For the outcome of asthma by age 5 years, we accounted for all residential addresses until age 3 years. 

### 2.3. Outcome Assessment

The recurrent wheeze outcome was derived using parent-reported data from parent interviews in which they were asked to provide detailed information regarding all of the child’s breathing problems, from birth to age 3 years. Recurrent wheeze by age 3 years was defined by parental report of at least 2 corticosteroid-requiring breathing problems in 6 months or at least 4 breathing problem episodes in 1 year that last at least 1 day and affected sleep [1,32]. Asthma by age 5 years was defined using parent-reported clinician diagnosis by the age of 5 years with either asthma medication use or asthma symptoms during the age of 4–4.9 years [33]. Additionally, data on general demographics, medical history, and family history of asthma, were also collected at time of enrollment. 

### 2.4. Statistical Analyses

Participants’ characteristics were described overall and by primary and secondary exposure to TRAP using counts and proportions, with bivariate associations tested using chi-square and Fisher’s exact test, as appropriate. Bivariate analysis of TRAP exposures and recurrent wheeze by age 3 years used log rank test of equality for continuous exposures and chi-square and Fisher’s exact test, as appropriate, for categorical exposures. Bivariate analysis of TRAP exposures and asthma by age 5 years used Wilcoxon rank sum for continuous exposures and used chi-square and Fisher’s exact test, as appropriate, for categorical exposures.

We used multivariable Cox proportional hazard modeling to assess the association between proximity to major road and primary road with recurrent wheeze by age 3 years. Time-to-event was calculated in days from birth to the date when the recurrent wheeze outcome definition was met. Participants were censored at the date of their 3-year follow-up interview or at their last follow-up interview. The models were stratified by sex and the results were reported as hazard ratios (HRs) with 95% CI. We used multivariable logistic regression to assess the association between the primary exposures and asthma by age 5 years. The models accounted for potential clustering by sites using a clustered sandwich estimator for standard errors, and the results were reported as odds ratios (ORs) with 95% CI. All multivariable models were adjusted for sex, race/ethnicity, enrollment age (dichotomized by age 2 months), prematurity (gestational age < 37 weeks), medical insurance type, median household income estimated using ZIP code at enrollment, parental history of asthma, population at the birth residential area, and infant tobacco exposure at enrollment. Covariates were selected based on relevance to the TRAP exposures and respiratory outcomes of our study, with the goal of creating parsimonious models. Additionally, we conducted sensitivity analyses using multivariable logistic regression to model road exposures with two combination outcomes: recurrent wheeze by age 3 years without asthma by age 5 years and recurrent wheeze by age 3 years with asthma by age 5 years. For both outcomes, non-cases were defined as having neither recurrent wheeze nor asthma.

Additionally, we plotted the distances between birth residence to major roads and pooled distances to major roads by incidence rates of recurrent wheeze by age 3 years. Incidence rates of recurrent wheeze were obtained from the summary statistics of the survival model for each of birth residence distance to major road and pooled distance to major road. Then we multiplied incidence rates by 100,000 and excluded distances >4000 m from each plot. All analyses were performed using Stata 14.1 (Stata Corp, College Station, TX, USA). All the *p*-values were two-tailed, with *p* < 0.05 considered statistically significant. Using cartographic tools of ArcGIS Pro 2.7 (ESRI, Redlands, CA, USA), we created maps depicting participants’ locations at enrollment by population density (2010 US Census) and ZIP code tabulation area, and by distribution of major roads and primary roads.

## 3. Results

### 3.1. Study Population

**Figure 1:**
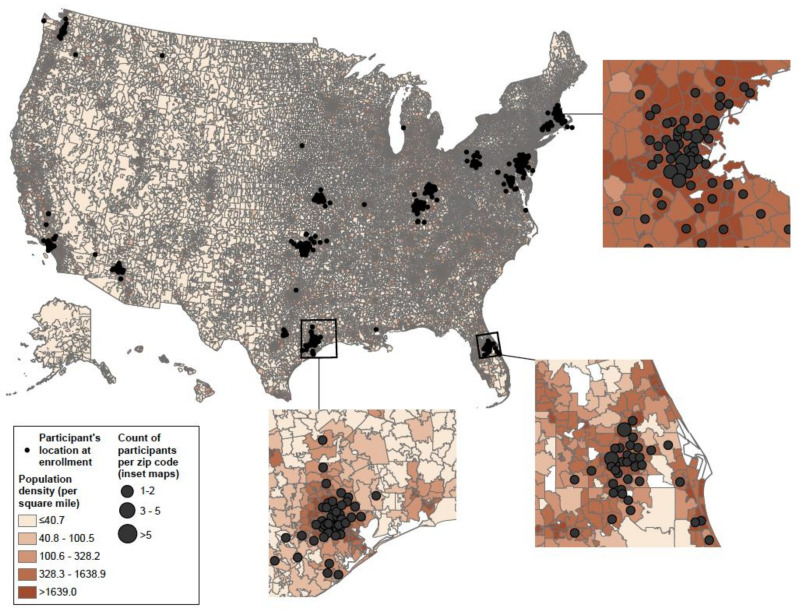
Distribution of participants’ enrollment addresses across the U.S. and distribution of population density obtained from 2010 census plotted by ZIP code tabulation areas.

Of 1016 infants enrolled in MARC-35 at baseline, 921 (91%) participated in the longitudinal data collection period. One participant was excluded from the analytical cohort because they only provided a PO Box number as the contact address during the entire study period. Among the remaining participants (*n* = 920), the majority were enrolled at age ≥ 2 months (71%), male (60%), had non-private health insurance (60%), and no parental history of asthma (67%) (Table 1). At the time of the study, the majority of our participants lived in densely populated areas spread across the U.S. (Figure 1).

### 3.2. Exposure Results

The majority of participants had a pooled residential location away from major roads: 58 (6%) were located within <100 m, 82 (9%) were 100–200 m, 101 (11%) were 201–300 m, and 679 (74%) were >300 m from a major road. Participant characteristics were similar across pooled proximity to major road categories, but non-Hispanic black participants were more likely to live within ≤200 m of a major road compared to other participants. Pooled proximity to primary roads had a similar distribution compared to major roads, with 34 (4%) located within <400 m, 93 (10%) were 400–1000 m, and 793 (86%) were >1000 m from a primary road (Table 2). A significantly higher proportion of non-Hispanic black and Hispanic participants had pooled distances 400–1000 m from primary roads and participants with non-private health insurance also had a higher proportion of pooled distances within 400–1000 m to primary road. Participants with a median household income estimated by ZIP at enrollment below $50,000 tended to have pooled distances ≤1000 m from primary roads.

### 3.3. Outcome Results

Among participants in the analytical cohort, 296 (32%) had developed recurrent wheeze by age 3 years (Table 3). By the time recurrent wheeze criteria had been met or by age 3 years for non-cases, 329 (36%) participants had moved, with a median length of stay at each address of 548 days and a median number of addresses of 1. Among participants who had developed recurrent wheeze by age 3 years, 65 (22%) had moved prior to reaching the outcome, with median length of stay of 337 days at each address and a median number of addresses of 1. Compared to participants without recurrent wheeze, those with recurrent wheeze had a higher pooled major road density within 250 m of radius circular area from all residential address (4.0 vs. 5.0 km roadway/km^2^). In addition, compared to non-cases, those who developed recurrent wheezing by age 3 years had borderline significantly higher proportions for birth residential distances within <100 m of a major road (8% vs. 13%) and pooled residential addresses with distance <100 m to major road (6% vs. 11%).

Among participants in the analytical cohort, 858 (92%) had available data to identify the asthma by age 5 years outcome (Table 4). Among participants with asthma outcome data, 235 (27%) had asthma by age 5 years. Overall, by age 3 years, 413 (48%) participants in long-term follow up had moved, with median length of stay at each address of 1095 days and median of 1 address. Among participants with asthma by age 5 years, 125 (53%) of them had moved. Compared to participants without asthma, those with asthma had a significantly shorter length of stay at each address (548 vs. 1095 days), a larger number of median moves (2 vs. 1 move), shorter distance to major road from birth residential addresses (425 vs. 601 m) and pooled residential addresses with distance to major roads (507 vs. 651 m). In addition, compared to non-cases, those who developed asthma had borderline significantly higher proportions for birth residence distances to major roads within <100 m (9% vs. 11%) and pooled residential addresses with distance < 100 m to major roads (6% vs. 9%).

Examining associations between road exposures and recurrent wheeze by age 3 years using unadjusted survival models, distance to major roads from birth residence of <100 m (HR: 1.53, 95% CI: 1.08–2.16) and pooled residential addresses with distance to major road <100 m (HR: 1.72, 95% CI: 1.18–2.50) had a significantly higher risk of recurrent wheeze by age 3 years compared to residences >300 m from major roads (Table 5). These associations were attenuated but remained in adjusted survival models, with distance to major road from birth residence of <100 m (HR: 1.43, 95% CI: 1.00–2.04) and pooled residential addresses with distance to nearest major road of <100 m (HR: 1.59, 95% CI: 1.08–2.33) being associated with increased risk for recurrent wheeze by age 3 years. For both birth addresses and pooled addresses, the intermediate distance to major road categories of 100–200 m and 201–300 m were not associated with an increased risk for recurrent wheeze compared to residences >300 m to major roads, suggesting a threshold of <100 m. The figures of recurrent wheeze incidence rate plotted against distance to major road from birth residence and pooled distance to major road from all residences, after multiply incidence rate with 100,000 and dropped road exposure >4000 m suggested linear relationships (Figures S1 and S2).

Examining associations between road exposures and asthma by age 5 years using unadjusted logistic regression models, distance to major roads from birth residences within 201–300 m (OR: 1.77, 95% CI: 1.11–2.82) and pooled residential addresses with distance to major roads <100 m (OR: 1.69, 95% Cl: 1.09–2.61) and within 201–300 m (OR: 1.62, 95% Cl: 1.10–2.39) had a significantly higher odds of developing asthma by age 5 years compared to residences >300 m from major roads (Table 6). In adjusted logistic regression models, pooled residential addresses with distance to major road <100 m (OR: 1.51, 95% Cl: 1.00–2.28) and within 201–300 m (OR: 1.62, 95% Cl: 1.16–2.25) remained associated with increased odds of asthma by age 5 years, while the association with birth residence distance to major road <100 m became attenuated.

Additionally, the sensitivity analysis using the combined outcome of recurrent wheeze by age 3 years with asthma by age 5 years had attenuated findings for distance to major roads <100 m from birth residence (OR: 1.65, 95% CI: 0.91–2.96), and pooled distance to major road <100 m (OR: 1.50, 95% CI: 0.94–2.40) and within 201–300 m (OR: 1.54, 95% CI: 0.95–2.48) (Table S2).

## 4. Discussion

### 4.1. Overall Findings

In this multicenter prospective cohort study, participants living within close birth and pooled proximity (<100 m) to major roads had higher risk of developing recurrent wheeze by age 3 years compared to those living >300 m away from their birth address. Risk of recurrent wheeze diminished as distance to major roads increased, suggesting a linear relationship with recurrent wheeze. Similarly, proximity to major roads of <100 m and 201–300 m to major roads from pooled address had higher odds of asthma by age 5 years. In addition, there was significant association between birth distance to major roads <100 m and asthma by age 5 years in an unadjusted model; however, the significance diminished after adjustment for covariates. There was no association between birth or pooled proximity to primary roads and risk of recurrent wheeze or asthma outcomes. As such, our results show that proximity to major roads, more than primary roads, have a significant association with the development of childhood airway disease. The current study highlights a knowledge gap in assessing TRAP exposure in high-risk populations, shows varying associations between TRAP and wheeze and asthma, and suggests that there are potential synergistic effects on health from TRAP proximity.

### 4.2. High-Risk Cohort Comparison

A distinct feature of our study is that we assessed exposures within a pediatric population in which all participants had a bronchiolitis episode in infancy that resulted in hospitalization. From previous literature, it is well established that infants with bronchiolitis represent a high-risk group for development of wheeze and asthma, and bronchiolitis is a common viral respiratory illness in infants [1,24,34]. Only one other study assessed bronchiolitis as an exposure in regards to TRAP, wheeze, and asthma [24]. A 2018 prospective cohort study using data from Children’s Health and Environmental Research (CHEER) in Korea analyzed children with a history of bronchiolitis found that closer residential proximity (<75 m) to a main road has significant higher risk of newly diagnosed asthma, and road length (>500 m) metric has significant higher risk of new wheeze [24]. The CHEER study found positive associations between residential proximity to TRAP and risk of recurrent wheeze and asthma among high-risk participants, though this was only statistically significant for asthma. In addition, it concludes that bronchiolitis is an effect modifier for asthma pathogenesis and early-life exposure to TRAP and history of infantile bronchiolitis can act synergistically to increase the risk of childhood asthma [24].

CHEER study researchers also observed an exposure-response relationship among all children, with and without a history of bronchiolitis, aged 6–14 years living within 700 m of a main road [24]. Researchers found positive linear trend of increasing proximity and odds of new onset of asthma among all (general) participants [24]. On the other hand, a positive exposure-response relationship for wheezing (ever) was observed, though not significant. By contrast, while our models did not test for trend, we observed a clear threshold in distance for recurrent wheeze but not asthma. However, a direct comparison between the CHEER study dose-response relationships and our categorical exposures to assess linear relationships cannot be made. Further, it is difficult to accurately compare our results to the CHEER data since exposure-responses were only assessed in the general group.

### 4.3. General Population Comparison

In general-pediatric population studies that assessed risk of wheeze (current, historical, or recurrent) or asthma and residential proximity exposure to TRAP, results have been inconsistent and often found weak or no association [15,16,17,18,19,35,36,37,38,39]. The Cincinnati Childhood Allergy and Air Pollution Study (CCAAPS) study found that infants (<1 year of age) living within 100 m of a moving traffic sources (e.g., highways) at birth did not have significant higher risk of recurrent wheeze [19]. Other CCAAPS study also pointed that using a single address to estimate environmental exposures over time resulted in exposure misclassification and may infer in differential errors [25]. Similarly, researchers of a birth cohort study conducted in New York City found that proximity to highways (<250 m) was not statistically significant for wheeze in all children by age 5 years. On the other hand, The New York City cohort analyzed asthma outcomes in which they found that proximity to highways was significant for asthma in children that have never moved by age 5 years. After assessing cumulative exposure (pooled residential distance) to major roads in our model by age 3 years, we found conflicting results for wheeze but not asthma: proximity to major roads was significantly associated with recurrent wheeze and asthma. We also found conflicting results analyzing only birth addresses and risk for recurrent wheeze: birth distance to major roads is significantly associated with increased risk of recurrent wheeze but not asthma after adjusting for confounders. Thus, our study shows that the cumulative TRAP exposure during childhood has differing effects on recurrent wheeze and asthma outcomes compared to general populations, regardless of assessing singular or limited time points in infancy.

In other cohorts that assessed long-term exposure, The German Infant Nutritional Intervention (GINI) study and the Influences of Life Style and Related Factors on the Immune System and the Development of Allergies in East and West Germany (LISA) cohorts found a significantly higher risk of wheeze and asthmatic/spastic/obstructive bronchitis from proximity to a main road (<50 m) exposed at birth, 2–3 years, and 6 years of age [20,21]. A marginally significant distance-dependent relationship in children living close to a main road and wheeze and asthma/asthmatic/spastic/obstructive bronchitis was found [20,21]. Similarly, a 2006 childhood cohort among southern California children aged 5–7 years found residential proximity of <75 m to a major road significantly increased the risk of wheeze, and prevalent and lifetime risk of asthma [36]. Additionally, a significant dose-response relationship was observed from decreasing proximity to major roads and increased risk for current wheeze and prevalent asthma [36]. Additionally, children who had not moved since 2 years of age (i.e., “lifetime residents”) had the highest risk of lifetime asthma, prevalent asthma, and recent wheeze from living close to a major road (<75 m). Though a linear relationship was observed among the lifetime residents, it was not statistically significant [36]. Our trends are similar, representing that linear associations remain inconsistent and variable when representing TRAP proximity and wheeze or asthma.

### 4.4. Synergistic Effects of TRAP

Our findings may also represent a possible association between synergistic TRAP effects and risk of respiratory disease. Our adjusted estimates for recurrent wheeze at pooled distance to major roads are higher compared to other studies that focused only on exposure to a primary pollutant and airway disease: where risk elevation is generally slightly significant in ranges of 3–20% [11,34]. Although relevant, the risk range for singular pollutants are results from meta-analyses that should be interpreted with caution, as they were unable to harmonize exposure assessment, outcomes, and confounders leading to substantial variability across studies [11,34]. Researchers of a 2017 systematic review later reported that results should be refined and that only a few studies assessed different and/or multiple exposure timeframes [40]. Additionally, it is well established that pollutants from various vehicles are at elevated concentrations closer to major roads [41,42]. We can infer that analyzing certain pollutants may provide insight for overall risk, there is inherently a complex mixture of air pollutants associated with traffic. Our study represents that the possible synergistic effects of TRAP source proximity exposure on development of recurrent wheeze and asthma may be higher than assessing just one air pollutant, and reinforces the established literature that there are higher concentrations of pollutants near traffic sources.

### 4.5. Study Strengths and Limitations

The present study has potential limitations. Our study population is higher risk for both recurrent wheeze and asthma, which limits generality of the results to all children. Conversely, our results represent the challenges of multiple environmental risk factors and the advancement of airway disease in a susceptible population. Additionally, we measured TRAP exposure by using proximity to TRAP sources (i.e., major and primary roads) as a surrogate. As such, our exposures did not account for specific air pollutants by measuring or modeling TRAP concentrations. However, evidence from multiple studies have shown that living within proximity to roads creates an exposure zone for a combination of TRAP and that pollutants from various vehicles are at elevated concentrations near major roads [41,42]. Thus, using proximity to major roads is suggested as a valuable approach in quantitating TRAP exposures and offers the availability to take into consideration a mixture of pollutants at once [41]. Our study was also reinforced by analyzing cumulative effects of TRAP exposure through residential proximity in varying degrees.

Our study was strengthened by the population scope that including multiple major cities throughout the U.S, availability of detailed address histories due to biannual follow up interviews and confirming addresses through direct contact with parents/guardians, low attrition rate, and robust outcome data. Our exposure measurements highlight the importance of the first three years of life when assessing environmental exposures and their influence on respiratory function later in childhood. Results on any synergistic effects should be interpreted with caution and future studies are needed to assess a long-term cumulative TRAP effects on risk of recurrent wheeze and asthma through a combination of TRAP source proximity, concentration, and land use modeling. Furthermore, additional long-term and multifactorial exposure analyses on sensitive populations would be beneficial to add to the weight of evidence suggesting TRAP exposures are associated with respiratory illness.

## 5. Conclusions

Our main results show that proximity to major roads increases risk of both recurrent wheeze and asthma in a high-risk pediatric cohort. Our findings are comparable to studies focusing on early-life TRAP exposure, adding to evidence that exposures surrounding birth affect respiratory health of a growing infant [2,5,11,12,13,14,15,16,17,18]. Ongoing investigation is needed to identify similar risks in other susceptible populations to the adverse effects of TRAP with the potential to include other environmental factors that may act synergistically. These findings support efforts to restrict residential construction within proximity of major roads and other sources of TRAP. While the association between air pollution exposure and the exacerbation and development of airway disease remain variable, our study adds to the body of literature emphasizing the importance of investigating cumulative early childhood exposure to air pollution and its potential to affect respiratory function later in life.

## Figures and Tables

**Table 1:** Participant characteristics by categories of pooled proximity to major roads before age 3 years.

Characteristics	Total (*n* = 920), *n* (%)	Pooled Proximity to Major Road before Age 3 Years, Meters				
		<100 (*n* = 58), *n* (%)	100–200 (*n* = 82), *n* (%)	201–300 (*n* = 101), *n* (%)	>300 (*n* = 679), *n* (%)	*p*-Value *
Age at enrollment, months						0.19
<2	271 (29)	19 (33)	16 (20)	33 (33)	203 (30)	
≥2	649 (71)	39 (67)	66 (80)	68 (67)	476 (70)	
Sex						0.18
Male	552 (60)	29 (50)	44 (54)	65 (64)	414 (61)	
Female	368 (40)	29 (50)	38 (46)	36 (36)	265 (39)	
Race/ethnicity						<0.001
Non-Hispanic white	400 (43)	19 (33)	30 (37)	48 (47)	303 (45)	
Non-Hispanic black	210 (23)	20 (34)	33 (40)	26 (26)	131 (19)	
Hispanic	275 (30)	15 (26)	17 (21)	27 (27)	216 (32)	
Other	35 (4)	4 (7)	2 (2)	0 (0)	29 (4)	
Health insurance						0.46
Non-private (e.g., public or none)	547 (60)	40 (69)	46 (58)	62 (61)	399 (59)	
Private	371 (40)	18 (31)	34 (42)	39 (39)	280 (41)	
Median household income estimated by ZIP code at enrollment						0.93
<$50,000	481 (52)	32 (55)	41 (50)	54 (53)	354 (52)	
≥$50,000	439 (48)	26 (45)	41 (50)	47 (47)	325 (48)	
Mother’s age at enrollment, years						0.51
<25	289 (31)	23 (40)	28 (34)	29 (29)	209 (31)	
25–29	263 (29)	12 (20)	24 (30)	34 (34)	193 (29)	
30–34	218 (24)	11 (19)	15 (18)	20 (20)	172 (25)	
≥35	148 (16)	12 (21)	15 (18)	17 (17)	104 (15)	
Mode of delivery						0.59
Vaginal delivery	596 (66)	41 (73)	56 (68)	64 (65)	435 (65)	
Cesarean section	312 (34)	15 (27)	26 (32)	34 (35)	237 (35)	
Premature birth (gestational age ≤37 weeks)	170 (18)	13 (22)	13 (16)	21 (21)	123 (18)	0.71
Infant tobacco exposure at enrollment	138 (15)	3 (5)	13 (16)	19 (19)	103 (15)	0.13
Parental history of asthma	305 (33)	19 (33)	29 (35)	32 (32)	225 (33)	0.91
Parental history of eczema	175 (19)	18 (31)	15 (18)	21 (21)	121 (18)	0.09

* All of the *p*-values obtained by excluding participants with any missing data.

**Table 2:** Participant characteristics by categories of pooled proximity to primary roads before age 3 years.

Characteristics	Polled Proximity to Primary Road before Age 3 Years, Meters			
	<400 (*n* = 34), *n* (%)	400–1000 (*n* = 93), *n* (%)	>1000 (n = 793), n (%)	*p*-Value *
Age at enrollment, months				0.25
<2	13 (38)	22 (24)	236 (30)	
≥2	21 (62)	71 (76)	557 (70)	
Sex				0.77
Male	20 (59)	59 (63)	473 (60)	
Female	14 (41)	34 (37)	320 (40)	
Race/ethnicity				0.02
Non-Hispanic white	13 (38)	27 (29)	360 (45)	
Non-Hispanic black	8 (24)	32 (34)	170 (22)	
Hispanic	12 (35)	33 (36)	230 (29)	
Other	1 (3)	1 (1)	33 (4)	
Health insurance				0.02
Non-private (e.g., public or none)	18 (55)	68 (73)	461 (58)	
Private	15 (45)	25 (27)	331 (42)	
Median household income estimated by ZIP code at enrollment				0.001
<$50,000	25 (74)	60 (65)	396 (50)	
≥$50,000	9 (26)	33 (35)	397 (50)	
Mother’s age at enrollment, years				0.23
<25	13 (38)	38 (41)	238 (30)	
25–29	9 (26)	26 (28)	228 (29)	
30–34	5 (15)	20 (21)	193 (24)	
≥35	7 (21)	9 (10)	132 (17)	
Mode of delivery				0.72
Vaginal delivery	23 (68)	63 (69)	510 (65)	
Cesarean section	11 (32)	28 (31)	273 (35)	
Premature birth (gestational age ≤ 37 weeks)	6 (18)	19 (20)	145 (18)	0.87
Infant tobacco exposure at enrollment	3 (9)	18 (19)	117 (15)	0.30
Parental history of asthma	11 (32)	35 (38)	259 (33)	0.70
Parental history of eczema	4 (12)	21 (23)	150 (19)	0.49

* All of the *p*-values obtained by excluding participants with any missing data.

**Table 3:** Distribution of road exposures at birth and pooled road exposures by recurrent wheeze status by age 3 years.

Road Exposures		Total (*n* = 920)	Without Recurrent Wheeze by Age 3 Years (*n* = 624)	With Recurrent Wheeze by Age 3 Years (*n* = 296)	*p*-Value *
Number of participants who moved, *n* (%)		329 (36)	264 (42)	65 (22)	<0.001
Number of moves per participant, median (IQR)		1 (1–2)	1 (1–2)	1 (1–1)	<0.001
Average length of stay in each address in days, median (IQR)		548 (355–1095)	559 (428–1095)	337 (233–494)	<0.001
Birth distance to major roads in meter, median (IQR)		550 (234–1135)	576 (240–1159)	474 (207–1067)	0.30
Pooled distance to major roads in meter, median (IQR)		565 (257–1141)	583 (274–1162)	526 (238–1078)	0.31
Birth distance to primary roads in meters, median (IQR)		3004 (1290–6273)	3018 (1277–6326)	2969 (1333–6206)	0.97
Pooled distance to primary roads in meters, median (IQR)		3266 (1391–7189)	3345 (1396–7522)	3120 (1388–6911)	0.76
Birth major road density in km roadways/km^2^ land area, median (IQR) †		5 (4–9)	5 (4–9)	5 (4–9)	0.63
Pooled major road density in km roadways/km^2^ land area, median (IQR) †,§		4 (3–7)	4 (2–7)	5 (3–8)	0.04
Birth primary road density in km roadways/km^2^ land area, median (IQR) †		6 (3–11)	6 (3–9)	11 (3–17)	0.14
Pooled primary road density in km roadways/km^2^ land area, median (IQR) †,§		4 (2–7)	4 (2–7)	4 (4–14)	0.07
Birth population density in population/mile^2^ land area, median (IQR) ‡		4456 (1795–10,756)	4174 (1560–10,744)	5068 (2066–11,325)	0.64
Pooled population density in (population/mile^2^ land area, median (IQR) ‡,§		4546 (1935–10,100)	4486 (1896–10,047)	5147 (2057–10,603)	0.93
Birth distance to major roads, meters	<100	85 (9)	47 (8)	38 (13)	0.07
	100–200	116 (13)	82 (13)	34 (12)	
	201–300	86 (9)	61 (10)	25 (8)	
	>300	632 (69)	433 (70)	199 (67)	
Birth distance to primary roads, meters	<400	59 (6)	45 (7)	14 (5)	0.34
	400–1000	114 (12)	78 (13)	36 (12)	
	>1000	746 (81)	500 (80)	246 (83)	
Pooled distance to major roads, meters §	<100	69 (8)	37 (6)	32 (11)	0.06
	100–200	95 (10)	63 (10)	32 (11)	
	201–300	101 (11)	69 (11)	32 (11)	
	>300	655 (71)	455 (73)	200 (67)	
Pooled distance to primary roads, meters §	<400	46 (5)	33 (5)	13 (4)	0.81
	400–1000	103 (11)	71 (12)	32 (11)	
	>1000	771 (84)	520 (83)	251 (85)	

Abbreviations: IQR = interquartile range. * All of the *p*-values obtained by excluding participants with any missing data. † Geographic information systems (GIS) based road measurements were done in a circular buffer area with a 250-radius surrounding the participants’ residential area. ‡ Population density of residential census block was assigned to each participant by weighted block centroid geographic retrieval. § Pooled estimates derived by weighting exposures according to duration spent each address up to age three or time outcome occurred, whichever happened first.

**Table 4:** Distribution of road exposures at birth and pooled road exposures by asthma status by age 5 years.

Road Exposures		Total (*n* = 858)	without Asthma by Age 5 Years (*n* = 623)	with Asthma by Age 5 Years (*n* = 235)	*p*-Value *
Number of participants who moved, *n* (%)		413 (48)	288 (46)	125 (53)	0.07
Number of moves per participant, median (IQR)		1 (1–2)	1 (1–2)	2 (1–2)	0.04
Average length of stay in each address in days, median (IQR)		1095 (548–1095)	1095 (548–1095)	548 (548–1095)	0.05
Birth distance to major roads in meter, median (IQR)		540 (236–1121)	601 (253–1177)	425 (211–960)	0.006
Pooled distance to major roads in meter, median (IQR)		601 (285–1199)	651 (305–1218)	507 (233–1082)	0.005
Birth distance to primary roads in meter, median (IQR)		3004 (1283–6275)	3167 (1301–6398)	2500 (1197–5885)	0.15
Pooled distance to primary roads in meter, median (IQR)		3430 (1491–7765)	3558 (1516–8429)	2908 (1483–6574)	0.14
Birth major road density in km roadways/km^2^ land area, median (IQR) †		5 (4–9)	5 (4–9)	6 (4–10)	0.61
Pooled major road density in km roadways/km^2^ land area, median (IQR) †,§		4 (2–7)	4 (2–6)	4 (2–8)	0.58
Birth primary road density in km roadways/km^2^ land area, median (IQR) †		6 (3–11)	6 (3–8)	9 (3–21)	0.35
Pooled primary road density in km roadways/km^2^ land area, median (IQR) †,§		4 (2–6)	4 (2–6)	2 (1–8)	0.64
Birth population density in population/m^2^ land area, median (IQR) ‡		4432 (1817–10,611)	3954 (1698–9615)	5713 (2075–13,244)	0.01
Pooled population density in (population/mile^2^ land area, median (IQR) ‡,§		4590 (2008–9793)	4210 (1961–9004)	6256 (2203–12,776)	0.005
Birth distance to major roads, meters	<100	82 (10)	55 (9)	27 (11)	0.08
	100–200	105 (12)	75 (12)	30 (13)	
	201–300	81 (9)	51 (8)	30 (13)	
	>300	589 (69)	442 (71)	147 (63)	
Birth distance to primary roads, meters	<400	55 (6)	37 (6)	18 (8)	0.59
	400–1000	108 (13)	77 (12)	31 (13)	
	>1000	694 (81)	509 (82)	185 (79)	
Pooled distance to major roads, meters §	<100	55 (6)	35 (6)	20 (9)	0.08
	100–200	74 (9)	53 (8)	21 (8)	
	201–300	96 (11)	62 (10)	34 (14)	
	>300	633 (74)	473 (76)	160 (69)	
Pooled distance to primary roads, meters §	<400	31 (4)	22 (3)	9 (4)	0.83
	400–1000	87 (10)	61 (10)	26 (11)	
	>1000	740 (86)	540 (87)	200 (5)	

Abbreviations: IQR = interquartile range. * All of the *p*-values obtained by excluding participants with any missing data. † Geographic information systems (GIS) based road measurements were done in a circular buffer area with a 250-radius surrounding the participants’ residential area. ‡ Population density of residential census block was assigned to each participant by weighted block centroid geographic retrieval. § Pooled estimates derived by weighting exposures according to duration spent each address up to age three or time outcome occurred, whichever happened first.

**Table 5:** Unadjusted and adjusted multivariable survival model of road exposures for recurrent wheeze by age 3 years as outcome.

Road Exposures		Unadjusted HR (95% CI)	*p*-Value	Adjusted HR (95% CI) *	*p*-Value
Birth distance to major road, meter	<100	1.53 (1.08–2.16)	0.02	1.43 (1.00–2.04)	0.05
	100–200	0.96 (0.66–1.38)	0.81	0.91 (0.63–1.31)	0.60
	201–300	0.93 (0.61–1.41)	0.74	0.95 (0.62–1.44)	0.80
	>300	Ref	---	Ref	---
Birth distance to primary road, meter	<400	0.69 (0.41–1.19)	0.19	0.72 (0.42–1.24)	0.24
	400–1000	0.91 (0.64–1.29)	0.58	0.89 (0.62–1.27)	0.51
	>1000	Ref	---	Ref	---
Pooled distance to major road, meter †	<100	1.72 (1.18–2.50)	0.004	1.59 (1.08–2.33)	0.02
	100–200	1.21 (0.84–1.76)	0.31	1.14 (0.78–1.66)	0.51
	201–300	1.04 (0.71–1.51)	0.85	1.03 (0.71–1.50)	0.88
	>300	Ref	---	Ref	---
Pooled distance to primary road, meter †	<400	0.90 (0.52–1.57)	0.72	0.92 (0.53–1.62)	0.78
	400–1000	0.91 (0.63–1.31)	0.62	0.89 (0.61–1.29)	0.53
	>1000	Ref	---	Ref	---

Abbreviations: HR = hazard ratio; CI = confidence interval; Ref = reference category. * Adjusted for age at enrollment (dichotomized by age 2 months), race/ethnicity, health insurance type, preterm birth (with cut-off at 37 months), median household income estimated by ZIP code at enrollment, parental history of asthma, population density of residential area at birth, and infant tobacco exposure at enrollment, and was stratified by sex. † Pooled estimates derived by weighting exposures according to duration spent each address up to age three or time outcome occurred, whichever happened first.

**Table 6:** Unadjusted and adjusted logistic regression models of road exposures for asthma by age 5 years as outcome.

Road Exposures		Unadjusted OR (95%CI)	*p*-Value	Adjusted OR (95%CI) *	*p*-Value
Birth distance to major road, meter	<100	1.48 (0.98–2.22)	0.06	1.26 (0.81–1.95)	0.31
	100–200	1.20 (0.75–1.93)	0.44	0.95 (0.65–1.38)	0.78
	201–300	1.77 (1.11–2.82)	0.02	1.62 (0.92–2.85)	0.10
	>300	Ref	---	Ref	---
Birth distance to primary road, meter	<400	1.34 (0.54–3.30)	0.53	1.13 (0.53–2.41)	0.76
	400–1000	1.11 (0.81–1.52)	0.53	0.95 (0.64–1.39)	0.78
	>1000	Ref	---	Ref	---
Pooled distance to major road, meter †	<100	1.69 (1.09–2.61)	0.02	1.51 (1.00–2.28)	0.05
	100–200	1.17 (0.81–1.69)	0.40	0.85 (0.54–1.34)	0.49
	201–300	1.62 (1.10–2.39)	0.02	1.62 (1.16–2.25)	0.005
	>300	Ref	---	Ref	---
Pooled distance to primary road, meter †	<400	1.11 (0.33–3.71)	0.87	1.07 (0.39–2.95)	0.89
	400–1000	1.15 (0.80–1.65)	0.45	1.03 (0.69–1.55)	0.87
	>1000	Ref	---	Ref	---

Abbreviations: OR = odds ratio; CI = confidence interval; Ref = reference category. * Adjusted for sex, age at enrollment (dichotomized by age 2 months), race/ethnicity, health insurance type, preterm birth (with cut-off at 37 months), median household income estimated by ZIP code at enrollment, parental history of asthma, population density of residential area at birth, and infant tobacco exposure at enrollment, and clustering by site. † Pooled estimates derived by weighting exposures according to duration spent each address up to age three or time outcome occurred, whichever happened first.

## Data Availability

The data presented in this study are available on reasonable request from the corresponding author. The data are not publicly available due to privacy restriction.

## Supplementary Materials

The following are available online at https://www.mdpi.com/article/10.3390/ijerph18084197/s1, Table S1: Principal investigators at the 17 participating sites in MARC-35, Table S2: Adjusted logistic regression models of road exposures for combined outcomes of recurrent wheeze by age 3 years and asthma by age 5 years, Figure S1: Recurrent wheeze incidence rate per 100,000 people with distance to major road from birth address ≤4000 m, Figure S2: Recurrent wheeze incidence rate per 100,000 people with pooled distance to major road ≤4000 m, Figure S3: Distribution of major and primary roads across USA.

## References

[1] Dumas O., Hasegawa K., Mansbach J.M., Sullivan A.F., Piedra P.A., Camargo C.A. (2019). Severe bronchiolitis profiles and risk of recurrent wheeze by age 3 years. J. Allergy Clin. Immunol..

[2] Jiang X.Q., Mei X.D., Feng D. (2016). Air pollution and chronic airway diseases: What should people know and do?. J. Thorac. Dis..

[3] Lasso-Pirot A., Delgado-Villalta S., Spanier A.J. (2015). Early childhood wheezers: Identifying asthma in later life. J. Asthma Allergy.

[4] Wright A.L. (2002). Epidemiology of asthma and recurrent wheeze in childhood. Clin. Rev. Allergy Immunol..

[5] Teague W.G., Bayer C.W. (2001). Outdoor air pollution. Asthma and other concerns. Pediatr. Clin. N. Am..

[6] Mallol J., García-Marcos L., Solé D., Brand P., EISL Study Group (2010). International prevalence of recurrent wheezing during the first year of life: Variability, treatment patterns and use of health resources. Thorax.

[7] Paul S.P., Bhatt J.M. (2014). Preschool Wheeze is Not Asthma: A Clinical Dilemma. Indian J. Pediatr..

[8] Martinez F.D., Wright A.L., Taussig L.M., Holberg C.J., Halonen M., Morgan W.J. (1995). Asthma and wheezing in the first six years of life. The Group Health Medical Associates. N. Engl. J. Med..

[9] Zar H.J., Ferkol T.W. (2014). The global burden of respiratory disease-impact on child health. Pediatr. Pulmonol..

[10] Sherman C.B., Tosteson T.D., Tager I.B., Speizer F.E., Weiss S.T. (1990). Early childhood predictors of asthma. Am. J. Epidemiol..

[11] Bowatte G., Lodge C., Lowe A.J., Erbas B., Perret J., Abramson M.J., Matheson M., Dharmage S.C. (2015). The influence of childhood traffic-related air pollution exposure on asthma, allergy and sensitization: A systematic review and a meta-analysis of birth cohort studies. Allergy.

[12] Pérez-Yarza E.G., Moreno-Galdó A., Ramilo O., Rubí T., Escribano A., Torres A., Sardón O., Oliva C., Pérez G., Cortell I. (2015). Risk factors for bronchiolitis, recurrent wheezing, and related hospitalization in preterm infants during the first year of life. Pediatr. Allergy Immunol..

[13] Trasande L., Thurston G.D. (2005). The role of air pollution in asthma and other pediatric morbidities. J. Allergy Clin. Immunol..

[14] Schultz E.S., Gruzieva O., Bellander T., Bottai M., Hallberg J., Kull I., Svartengren M., Melén E., Pershagen G. (2012). Traffic-related air pollution and lung function in children at 8 years of age: A birth cohort study. Am. J. Respir. Crit. Care Med..

[15] Pfeffer P.E., Mudway I.S., Grigg J. (2021). Air Pollution and Asthma: Mechanisms of Harm and Considerations for Clinical Interventions. Chest.

[16] Bernstein D.I. (2012). Traffic-related pollutants and wheezing in children. J. Asthma.

[17] Patel M.M., Quinn J.W., Jung K.H., Hoepner L., Diaz D., Perzanowski M., Rundle A., Kinney P.L., Perera F.P., Miller R.L. (2011). Traffic density and stationary sources of air pollution associated with wheeze, asthma, and immunoglobulin E from birth to age 5 years among New York City children. Enviorn. Res..

[18] Oftedal B., Nystad W., Brunekreef B., Nafstad P. (2009). Long-term traffic-related exposures and asthma onset in schoolchildren in oslo, norway. Enviorn. Health Perspect..

[19] Ryan P.H., LeMasters G., Biagini J., Bernstein D., Grinshpun S.A., Shukla R., Wilson K., Villareal M., Burkle J., Lockey J. (2005). Is it traffic type, volume, or distance? Wheezing in infants living near truck and bus traffic. J. Allergy Clin. Immunol..

[20] Morgenstern V., Zutavern A., Cyrys J., Brockow I., Gehring U., Koletzko S., Bauer C.P., Reinhardt D., Wichmann H.E., Heinrich J. (2007). Respiratory health and individual estimated exposure to traffic-related air pollutants in a cohort of young children. Occup Enviorn. Med..

[21] Morgenstern V., Zutavern A., Cyrys J., Brockow I., Koletzko S., Krämer U., Behrendt H., Herbarth O., von Berg A., Bauer C.P. (2008). Atopic diseases, allergic sensitization, and exposure to traffic-related air pollution in children. Am. J. Respir. Crit. Care Med..

[22] Lau N., Norman A., Smith M.J., Sarkar A., Gao Z. (2018). Association between traffic related air pollution and the development of asthma phenotypes in children: A systematic review. Int. J. Chronic Dis..

[23] Ranzi A., Porta D., Badaloni C., Cesaroni G., Lauriola P., Davoli M., Forastiere F. (2014). Exposure to air pollution and respiratory symptoms during the first 7 years of life in an Italian birth cohort. Occup. Enviorn. Med..

[24] Lee J.Y., Leem J.H., Kim H.C., Lamichhane D.K., Hwang S.S., Kim J.H., Park M.S., Jung D.Y., Ko J.K., Kwon H.J. (2018). Effects of traffic-related air pollution on susceptibility to infantile bronchiolitis and childhood asthma: A cohort study in Korea. J. Asthma.

[25] Carlsten C., Dybuncio A., Becker A., Chan-Yeung M., Brauer M. (2011). Traffic-related air pollution and incident asthma in a high-risk birth cohort. Occup. Enviorn. Med..

[26] Girguis M.S., Strickland M.J., Hu X., Liu Y., Chang H.H., Belanoff C., Bartell S.M., Vieira V.M. (2017). Chronic PM_2.5_ exposure and risk of infant bronchiolitis and otitis media clinical encounters. Int. J. Hyg. Environ. Health.

[27] Hehua Z., Qing C., Shanyan G., Qijun W., Yuhong Z. (2017). The impact of prenatal exposure to air pollution on childhood wheezing and asthma: A systematic review. Environ. Res..

[28] Hasegawa K., Piedra P.A., Bauer C.S., Celedón J.C., Mansbach J.M., Spergel J.M., Espinola J.A., Camargo C.A., MARC-35 Investigators (2018). Nasopharyngeal CCL5 in infants with severe bronchiolitis and risk of recurrent wheezing: A multi-center prospective cohort study. Clin. Exp. Allergy.

[29] Gehring U., Wijga A.H., Brauer M., Fischer P., de Jongste J.C., Kerkhof M., Oldenwening M., Smit H.A., Brunekreef B. (2010). Traffic-related air pollution and the development of asthma and allergies during the first 8 years of life. Am. J. Respir. Crit. Care Med..

[30] Karner A.A., Eisinger D.S., Niemeier D.A. (2010). Near-roadway air quality: Synthesizing the findings from real-world data. Enviorn. Sci. Technol..

[31] Zhou Y., Levy J.I. (2007). Factors influencing the spatial extent of mobile source air pollution impacts: A meta-analysis. BMC Public Health.

[32] Geller R.J., Espinola J.A., Fabiano Filho R.C., Hasegawa K., Mansbach J.M., Sullivan A.F., Camargo C.A. (2021). A comparison of childhood asthma case definitions based on parent-reported data. Ann. Epidemiol..

[33] Gehring U., Wijga A.H., Hoek G., Bellander T., Berdel D., Brüske I., Fuertes E., Gruzieva O., Heinrich J., Hoffmann B. (2015). Exposure to air pollution and development of asthma and rhinoconjunctivitis throughout childhood and adolescence: A population-based birth cohort study. Lancet Respir. Med..

[34] Khreis H., Kelly C., Tate J., Parslow R., Lucas K., Nieuwenhuijsen M. (2017). Exposure to traffic-related air pollution and risk of development of childhood asthma: A systematic review and meta-analysis. Enviorn. Int..

[35] McConnell R., Berhane K., Yao L., Jerrett M., Lurmann F., Gilliland F., Künzli N., Gauderman J., Avol E., Thomas D. (2006). Traffic, susceptibility, and childhood asthma. Enviorn. Health Perspect..

[36] Jung D.Y., Leem J.H., Kim H.C., Kim J.H., Hwang S.S., Lee J.Y., Kim B.J., Hong Y.C., Hong S.J., Kwon H.J. (2015). Effect of Traffic-Related Air Pollution on Allergic Disease: Results of the Children’s Health and Environmental Research. Allergy Asthma Immunol. Res..

[37] Yi S.J., Shon C., Min K.D., Kim H.C., Leem J.H., Kwon H.J., Hong S., Kim K., Kim S.Y. (2017). Association between Exposure to Traffic-Related Air Pollution and Prevalence of Allergic Diseases in Children, Seoul, Korea. BioMed Res. Int..

[38] Gauderman W.J., Avol E., Lurmann F., Kuenzli N., Gilliland F., Peters J., McConnell R. (2005). Childhood asthma and exposure to traffic and nitrogen dioxide. Epidemiology.

[39] Buteau S., Doucet M., Tétreault L.F., Gamache P., Fournier M., Brand A., Kosatsky T., Smargiassi A. (2018). A population-based birth cohort study of the association between childhood-onset asthma and exposure to industrial air pollutant emissions. Enviorn. Int..

[40] Khreis H., Nieuwenhuijsen M.J. (2017). Traffic-related air pollution and childhood asthma: Recent advances and remaining gaps in the exposure assessment methods. Int. J. Enviorn. Res. Public Health.

[41] WHO Regional Office for Europe (2013). Review of Evidence on Health Aspects of Air Pollution—REVIHAAP Project: Technical Report [Internet].

[42] EPA Near Roadway Air Pollution and Health: Frequently Asked Questions. https://www.epa.gov/sites/production/files/2015-11/documents/420f14044_0.pdf.

