# Supplementary material for: Proximity to Major Roads and Risks of Childhood Recurrent Wheeze and Asthma in a Severe Bronchiolitis Cohort
Source: Int J Environ Res Public Health. 2021 Apr 15;18(8):4197. doi: 10.3390/ijerph18084197 (PMC8071463; doi:10.3390/ijerph18084197)
Supplement: Supplementary file 1 [file ijerph-18-04197-s001.zip › ijerph-1147311-supplementary.pdf]

International Journal of *Environmental Research and Public Health*

MDPI

Supplementary Materials

Table S1. Principal investigators at the 17 participating sites in MARC-35**.**

Amy D. Thompson, MD	Alfred I. duPont Hospital for Children, Wilmington, DE
Federico R. Laham, MD, MS	Arnold Palmer Hospital for Children, Orlando, FL
Jonathan M. Mansbach, MD, MPH	Boston Children's Hospital, Boston, MA
Vincent J. Wang, MD, MHA and Susan Wu, MD	Children's Hospital of Los Angeles, Los Angeles, CA
Michelle B. Dunn, MD and Jonathan M. Spergel, MD, PhD	Children's Hospital of Philadelphia, Philadelphia, PA
Juan C. Celedón, MD, DrPH	Children's Hospital of Pittsburgh, Pittsburgh, PA
Michael R. Gomez, MD, MS-HCA and Nancy R. Inhofe, MD	Children's Hospital at St. Francis, Tulsa, OK
Brian M. Pate, MD and Henry T. Puls, MD	Children's Mercy Hospital & Clinics, Kansas City, MO
Stephen J. Teach, MD, MPH	Children's National Medical Center, Washington, DC
Stephen C. Porter, MD, MSc, MPH and Richard T. Strait, MD	Cincinnati Children's Hospital and Medical Center, Cincinnati, OH
Ilana Y. Waynik, MD	Connecticut Children's Medical Center, Hartford, CT
Sujit S. Iyer, MD	Dell Children's Medical Center of Central Texas, Austin, TX
Ari R. Cohen, MD, Margaret Samuels-Kalow, MD, MPhil, MSHP and Wayne G. Shreffler, MD, PhD	Massachusetts General Hospital, Boston, MA
Michelle D. Stevenson, MD, MS	Norton Children’s Hospital and the University of Louisville, Louisville, KY
Cindy S. Bauer, MD and Anne K. Beasley, MD	Phoenix Children's Hospital, Phoenix, AZ
Markus Boos, MD, PhD and Thida Ong, MD	Seattle Children's Hospital, Seattle, WA
Charles G. Macias, MD, MPH and Sarah Meskill, MD	Texas Children's Hospital, Houston, TX

Table S2. Adjusted logistic regression models of road exposures for combined outcomes of recurrent wheeze by age 3 years and asthma by age 5 years.

Road exposures		Recurrent wheeze by age 3 years, without asthma by age 5 years		Recurrent wheeze by age 3 years, with asthma by age 5 years	
		Adjusted OR (95% CI)*	P-value	Adjusted OR (95% CI)*	P-value
Birth distance to major road, meter	<100	1.76 (0.82-3.79)	0.15	1.65 (0.91-2.96)	0.09
	100-200	0.90 (0.59-1.38)	0.64	0.80 (0.48-1.32)	0.38
	201-300	0.76 (0.32-1.79)	0.53	1.55 (0.73-3.30)	0.25
	>300	Ref	---	Ref	---
Birth distance to primary road, meter	<400	0.38 (0.12-1.19)	0.10	0.94 (0.58-1.53)	0.82
	400-1000	0.58 (0.30-1.12)	0.10	1.02 (0.56-1.85)	0.94
	>1000	Ref	---	Ref	---
Pooled distance to major road, meter†	<100	1.31 (0.55-3.10)	0.54	1.50 (0.94-2.40)	0.09
	100-200	0.79 (0.40-1.54)	0.49	0.68 (0.32-1.45)	0.32
	201-300	0.92 (0.41-2.05)	0.83	1.54 (0.95-2.48)	0.08
	>300	Ref	---	Ref	---
Pooled distance to primary road, meter†	<400	1 (empty)	---	0.43 (0.13-1.42)	0.17
	400-1000	0.52 (0.24-1.16)	0.11	0.99 (0.68-1.42)	0.93
	>1000	Ref	---	Ref	---

Abbreviations: OR, odds ratio; CI, confidence interval; Ref, reference category. * Adjusted for sex, age at enrollment (dichotomized by age 2 months), race/ethnicity, health insurance type, preterm birth (with cut-off at 37 months), median household income estimated by ZIP code at enrollment, parental history of asthma, population density of residential area at birth, and infant tobacco exposure at enrollment, and clustering by site. † Pooled estimates derived by weighting exposures according to duration spent each address up to age three or time outcome occurred, whichever happened first.

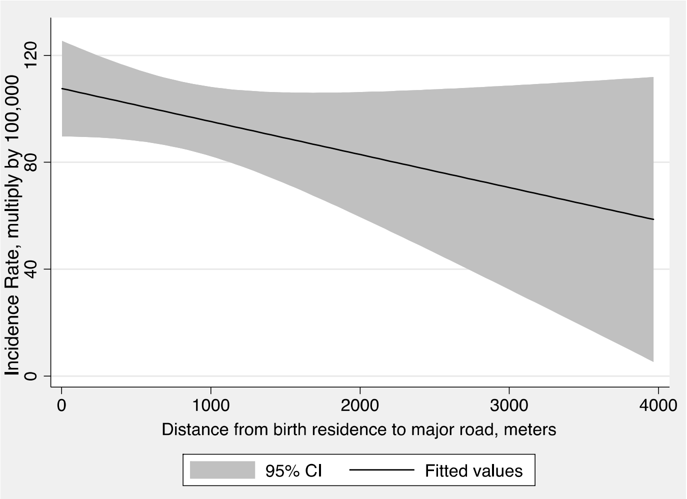

Figure S1: Recurrent wheeze incidence rate multiplied by 100,000 with distance to major road from birth address ≤4,000 meters

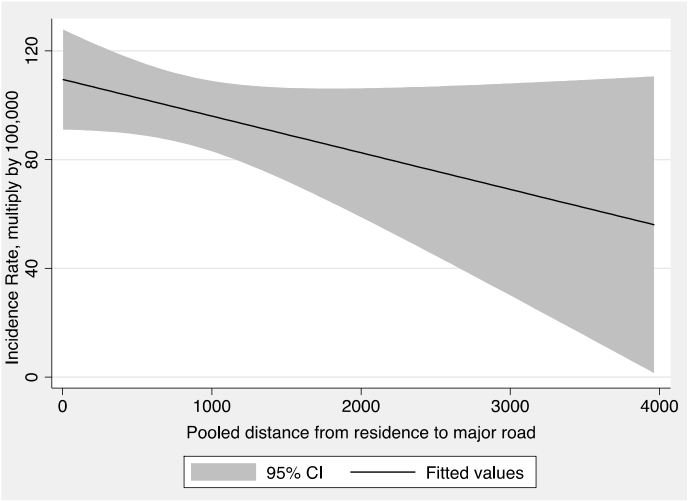

Figure S2: Recurrent wheeze incidence rate multiplied by 100,000 with pooled distance to major road ≤4,000 meters

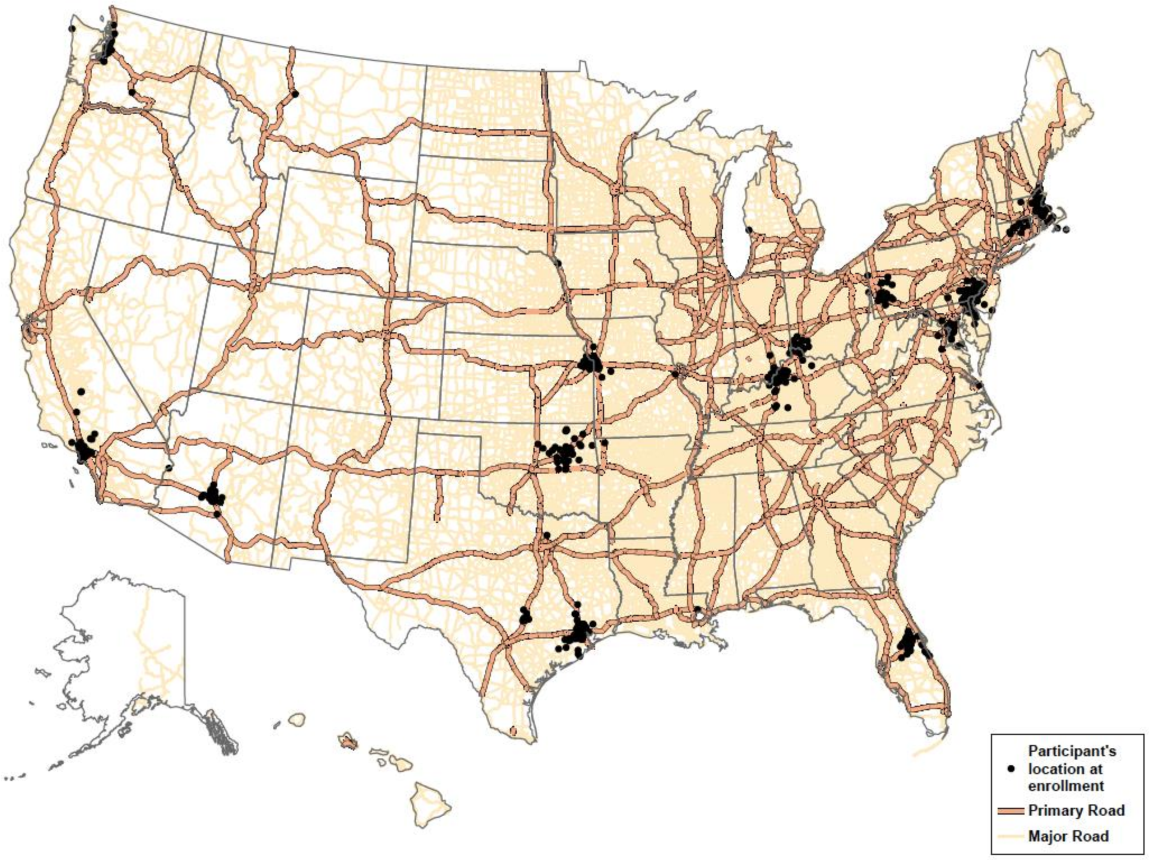

Figure S3: Distribution of major and primary roads across US
